# Less Is More: Higher-Skilled Sim Racers Allocate Significantly Less Attention to the Track Relative to the Display Features than Lower-Skilled Sim Racers

**DOI:** 10.3390/vision8020027

**Published:** 2024-04-29

**Authors:** John M. Joyce, Mark J. Campbell, Fazilat Hojaji, Adam J. Toth

**Affiliations:** 1 Esports Science Research Lab, Lero, The Science Foundation Ireland Centre for Software Research, University of Limerick, V94 T9PX Limerick, Ireland; john.m.joyce@ul.ie (J.M.J.); fazilat.hojaji@ul.ie (F.H.); adam.toth@ul.ie (A.J.T.); 2 Department of Physical Education & Sport Science, University of Limerick, V94 T9PX Limerick, Ireland; 3 Centre for Sport Leadership, Stellenbosch University, Stellenbosch 7602, South Africa

**Keywords:** esports, gaming, expertise, gaze behaviour, attentional control

## Abstract

Simulated (sim) racing is an emerging esport that has garnered much interest in recent years and has been a relatively under-researched field in terms of expertise and performance. When examining expertise, visual attention has been of particular interest to researchers, with eye tracking technology commonly used to assess visual attention. In this study, we examined the overt visual attention allocation of high- and low-skilled sim racers during a time trial task using Tobii 3 glasses. In the study, 104 participants were tested on one occasion, with 88 included in the analysis after exclusions. Participants were allocated to either group according to their fastest lap times. Independent *t*-tests were carried out with sidak corrections to test our hypotheses. Our results indicate that when eye tracking metrics were normalised to the lap time and corner sector time, there was a difference in the relative length of overt attention allocation (fixation behaviour) as lower-skilled racers had significantly greater total fixation durations in laps overall and across corner sectors when normalised (*p* = 0.013; *p* = 0.018). Interestingly, high- and low-skilled sim racers differed in where they allocated their attention during the task, with high-skilled sim racers allocating significantly less overt attention to the track relative to other areas of the display (*p* = 0.003). This would allow for higher-skilled racers to obtain relatively more information from heads-up display elements in-game, all whilst driving at faster speeds. This study provides evidence that high-skilled sim racers appear to need significantly less overt attention throughout a fast lap, and that high- and low-skilled sim racers differ in where they allocate their attention while racing.

## 1. Introduction

Simulated (sim) racing strives to recreate real-world motor racing experiences in a virtual environment [1]. Sim racing is one of the oldest esports and has recently gained much more popularity [2]. Tudor [3] notes how sim racing came to replace real-world motorsport during the COVID-19 pandemic with Formula (1)'s virtual grand prix series garnering approximately 7.5 million viewers over the course of eight events. When compared to real-world motorsport, sim racing lacks vestibular feedback as there are no g-forces acting upon the sim racer. Thus, in addition to noisy haptic feedback provided sometimes through their wheelbase and pedal set, sim racers must rely more on visual feedback to navigate their way along the virtual racetrack [4]. As a result of the high virtual speeds attained during sim racing, this visual feedback must be processed quickly, necessitating efficient visual attention behaviour.

Visual attention is important in many performance-based environments and the way visual attention is typically measured is through the use of eye tracking technology. The number of fixations, their duration and where they are allocated in one’s field of view all shed light on the amount and types of visual information processed by the observer [5]. These gaze behaviours have been used to differentiate expertise in many performance contexts, including surgery [6,7], medicine [8,9], and sport [10,11]. In sport, in particular, perceptual–cognitive skills are a feature that differentiates expert and novice performers, with experts demonstrating the ability to more rapidly process information in the environment and produce relevant movements for successful task completion [12]. For example, in a recent review by Brams and colleagues [13], it was found that in 10 out of the 19 studies examined, experts across various sports displayed a greater number of fixations at more locations (faster visual search rate) in comparison to intermediate- or novice-level performers. Interestingly, in the remaining visual search rate studies examined in the review, experts were found to have fewer fixations or fixations on a smaller number of locations (slower visual search rate). Therefore, it would seem that there is a lack of consensus on the visual behaviours of experts and novices, but the findings from the work by Vickers [14,15] that expert performers exhibit fewer and longer duration fixations in comparison to non-experts when performing self-paced tasks seem to suggest that the task type may contribute to these patterns.

Sim racing may be classified as either an externally paced or self-paced type of task depending on whether one is racing against opponents on the same track or performing a time trial, whereby they are attempting to achieve their fastest lap on an empty track. Race situations are very much externally paced. The actions of competitors must be anticipated and reacted to, and decisions need to be taken to initiate or defend overtakes. In contrast, during a practice or qualifying situation, the driver has time to prepare for a fast lap on their out lap (lap after exiting the pit garage), and during their fastest lap attempt, they dictate when to brake, turn in, and accelerate out of corners. Although numerous studies exist examining visual attention in real-world driving (e.g., [16]) and simulated driving tasks (e.g., [17,18]), no study has examined how visual attention is allocated by experts and novices in a simulated racing environment.

The purpose of this study is to determine if simulated racers of high and low skill overtly allocate their visual attention differently when completing a lap of a racetrack and across individual corner sectors. As we intend to examine a self-paced time trial, we firstly hypothesise that low-skilled sim racers will have greater attention allocation than high-skilled sim racers, evidenced by longer average and total fixation durations, and a greater number of fixations when unadjusted for their lap times (H1). Secondly, to mitigate the effect that a longer lap time may have on attention allocation opportunity, we also hypothesise that when metrics have been adjusted for their lap times, high-skilled sim racers will allocate attention for longer durations in fewer locations than low-skilled sim racers. This will be evidenced by high-skilled participants exhibiting lower fixations per second and higher average and total fixations durations as a proportion of lap time compared to low-skilled participants (H2). Thirdly, we hypothesise that high-skilled sim racers will attend more to task-relevant information (e.g., the track ahead; TRACK) relative to the supplementary on-screen information (e.g., heads up display (HUD) elements in-game) than low-skilled sim racers. This will be evidenced by a higher ratio of fixations (TRACK:HUD) on track for high-skilled sim racers compared to low-skilled sim racers (H3).

We also endeavoured to examine task-specific overt attention allocation across corners of the track. Previous research has found corner navigation to induce higher levels of cognitive processing while driving [19]. Therefore, fourthly, we hypothesise that that lower-skilled sim racers will have greater attention allocation than high-skilled sim racers, evidenced by longer average and total fixation durations, and a greater number of fixations in corner sectors when unadjusted for corner sector time (H4). Finally, we hypothesise that when metrics have been adjusted for corner sector time, high-skilled sim racers will allocate attention for longer durations in fewer locations than low-skilled sim racers. This will be evidenced by high-skilled participants exhibiting lower fixations per second, and higher average and total fixations durations as a proportion of corner sector time across all corners compared to low-skilled sim racers (H5).

## 2. Materials and Methods

### 2.1. Participants

A total of 104 participants (age = 30.84 ± 9.51) were recruited at a sim racing convention (ADAC Sim Racing Expo Nuremberg, 2022). Approval for the study was authorised by the research ethics board at the University of Limerick in accordance with the Declaration of Helsinki.

### 2.2. Materials

Two Playseat Sensation (Playseat, NL) sim racing setups with Logitech G Pro Wheelbases and Pedals (Logitech, CH) were used for data collection. Both racing setups were powered by PC and information was displayed on 55-inch (Samsung, Suwon-si, Republic of Korea) monitors with a refresh rate of 120 Hz. We used the racing title Assetto Corsa Competizione (v1.8.18) [20], in-game and the hardware and software settings can be found in Appendix A. MoTeC (v.1.1.5.0085) telemetry software [21] was used to capture participants telemetry data. To collect gaze data, participants wore Tobii Pro Glasses 3 [22] which sampled data at a rate of 50 Hz. The eye tracking glasses were connected to a separate PC where they could be controlled via the Tobii Pro Glasses 3 Controller app.

### 2.3. Protocol

Participants filled out a demographic questionnaire that also gathered information regarding their previous sim racing experience. Following this, participants were then instructed to get into the optimal seating position for themselves in one of the sim racing setups. They were then given the eye tracking glasses to wear and a one-point calibration was performed. This one-point calibration has been found to be accurate to within 1.60° [23], where accuracy refers to the angular distance from a target.

**Figure 1:**
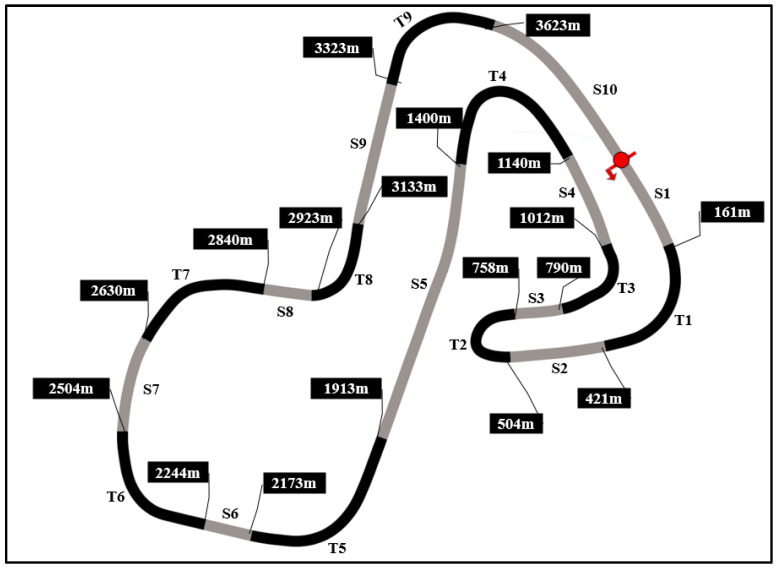
The Brands Hatch Circuit used in data collection. Figure shows the corner sectors used in the current study with corners seen in black. Adapted from Hojaji and colleagues [24].

Participants then drove 8 laps around the Brands Hatch circuit (Figure 1) using the McLaren 720s GT3 (setup on the car could not be changed) as fast as they could. Following the completion of their eighth lap, the eye tracking recording was stopped.

### 2.4. Data Processing

Following exclusions for incomplete eye tracking data, 88 participants remained. From this pool of participants, two groups were formed based on lap time. The fastest lap time each participant achieved was obtained from a review of eye tracking footage. Thereafter, we conducted a percentile split of the fastest lap times across all 88 participants, whereby the top 25% (high skilled) and bottom 25% (low skilled) lap times were segregated into two groups (*n* = 22 in each group).

In each participant’s eye tracking file, two events were inserted to mark the beginning and end of the gaze data corresponding to their fastest lap while reviewing the video footage of their recording using Tobii Pro Lab v1.207 [24]. Using these two events, a time of interest (TOI) was created which included all gaze data during a participant’s fastest lap.

**Figure 2:**
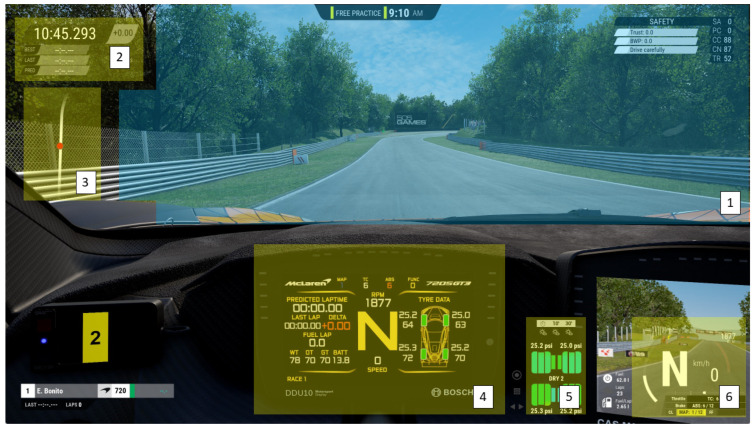
Snapshot image and the areas of interest (AOIs) for track (blue) (1) and HUD elements (yellow). HUD elements in the image include the lap time (2), circuit map (3), in-car information screen (4), tyre and brake temperatures (5), and speedometer (6).

To ascertain where participants were looking during their TOI, areas of interest (AOIs) were overlaid on a still image (Figure 2), and fixation mapping to the still image was applied for each participant. The assisted mapping feature in Tobii Pro Lab compares the snapshot image with each frame in the recording. The algorithm looks for similarities in the contrast and colour between the snapshot image and video recording. Gaze points are then automatically mapped from each frame to the snapshot according to a similarity score, where a similarity threshold can be set (in this case 50%), indicating how confident the algorithm is with the mapping. All automatically mapped points were reviewed and mapped manually if the fixation point in the video recording did not match the automatically mapped points following the assisted mapping. AOIs for the heads-up display (HUD), which included the lap time display, circuit map, in-car information screen, tyre and brake temperature display, and speedometer, and the whole view outside the car (i.e., the track ahead; TRACK) were created.

Before exporting the data from Tobii Pro Lab, the Tobii I-VT attention filter was applied to the data within the TOI. Further details on this filter can be found in Appendix B. The attention filter was used as it is recommended by the eye tracker manufacturer [25] (p. 172) when using eye tracking glasses under dynamic conditions. Thus, exported calculations were based off the attention filter. Only data within the TOI outlined above were exported for further analyses. The eye tracking data from participants’ fastest laps were also synchronised using timestamps from the corresponding telemetry data. This allowed us to examine eye tracking data corresponding specifically to corner sectors (defined according to Hojaji and colleagues [24] (Figure 1)).

Variables exported from Tobii Pro Lab for analysis included the following: total whole fixation duration (TFD; sum of all individual fixation durations across the fastest lap), average fixation duration (AFD; average duration of an individual fixation across all fixations for the fastest lap), fixation count (FC; total number of fixations across the fastest lap), these same variables normalised to each participant’s lap time (TFD_n_; AFD_n_; FC_n_), and the ratio of fixations allocated on the track vs. heads-up display (TRACK:HUD). When synchronised with the telemetry data, these metrics were calculated for each corner sector (Corner TFD; sum of all fixation durations within individual corners, Corner AFD; average duration of individual fixations across all fixations within individual corners, Corner FC; total number of fixations across all corners). In instances where the first fixation in a corner began at the end of the previous straight sector, this fixation and its corresponding duration were incorporated into the analysis for that corner. Where the last fixation in a corner sector extended into the following straight sector, this fixation was removed from the analysis of that corner. These variables were then normalised in relation to the time spent in individual corner sectors and then averaged (Corner TFD_n_; Corner AFD_n_; Corner FC_n_).

The calculations for each normalised metric and the TRACK:HUD metric are provided below.

*Normalised Total Fixation Duration (TFD_n_)*
(1) $${\mathrm{TFD}}_{n}=\frac{\mathrm{TFD}\;(\mathrm{ms})}{\mathrm{Lap}\;\mathrm{time}\;(\mathrm{ms})}\times 100$$
*Normalised Average Fixation Duration (AFD_n_)*
(2) $${\mathrm{AFD}}_{n}=\frac{\mathrm{AFD}\;(\mathrm{ms})}{\mathrm{Lap}\;\mathrm{time}\;(\mathrm{ms})}\times 100$$
*Fixations Per Second (FC_n_)*
(3) $${\mathrm{FC}}_{n\;}=\frac{\mathrm{FC}}{\mathrm{Lap}\;\mathrm{time}\;(\mathrm{ms})}\times 1000$$
*TRACK:HUD Fixation Ratio*
(4) $${\mathrm{FC}}_{\mathrm{TRACK}}:{\mathrm{FC}}_{\mathrm{HUD}}$$
*Average Fixations Per Second (FPS) across Corners (Corner FC_n_)*
  (5) $$\mathrm{Corner}\;{\mathrm{FC}}_{n}=\frac{\sum_{i=1}^{9}{\mathrm{CFPS}}_{i}}{9\;}$$
  where CFPS refers to the number of fixations in a corner divided by the total time spent in that corner sector. Average CFPS value calculated across all 9 corner sectors (Corner FC_n_).
*Normalised Average Fixation Duration across Corners (Corner AFD_n_)*
  (6) $$\mathrm{Corner}\;{\mathrm{AFD}}_{n}=\frac{\sum_{i=1}^{9}C{\mathrm{AFD}}_{\mathrm{ni}}}{9}$$
  where CAFD_n_ refers to the average fixation duration in a corner sector divided by the total time spent in that corner sector, multiplied by 100. Average CAFD_n_ value calculated across all 9 corner sectrors (Corner AFD_n_).
*Normalised Total Fixation Duration across Corners (Corner TFD_n_)*
  (7) $$\mathrm{Corner}\;{\mathrm{TFD}}_{n}=\frac{\sum_{i=1}^{9}{\mathrm{CTFD}}_{\mathrm{ni}}}{9}$$
  where CTFD_n_ refers to the total fixation duration in a corner sector divided by the total time spent in that corner sector, multiplied by 100. Average CTFD_n_ value calculated across all 9 corner sectors (Corner TFD_n_).

### 2.5. Data Analysis

Statistical analyses were carried out using SPSS v.28.0. After removing the outliers (fixations and average fixation durations exceeding 1.5 times the interquartile range; the data of 3 participants from the low skilled group were excluded), the Shapiro–Wilk test and the investigation of Q-Q plots were performed on dependent variables to verify the normality of the data.

To investigate whether low-skilled sim racers displayed greater overt attention allocation than high-skilled sim racers when metrics were unadjusted for lap time, we compared the fixation count, average fixation duration, and total fixation duration between high- and low-skilled sim racers using independent *t*-tests.

To investigate whether high-skilled sim racers displayed greater overt attention allocation when metrics were adjusted for the lap time, we compared fixations per second, average, and total fixation durations as a proportion of lap time between high- and low-skilled sim racers using independent *t*-tests.

To investigate whether high-skilled sim racers overtly attended to more task-relevant information, we compared TRACK:HUD fixations between the high- and low-skilled group using an independent *t*-test.

To investigate whether low-skilled sim racers displayed greater overt attention allocation than high-skilled sim racers in corner sectors when metrics were unadjusted for lap time, we compared the fixation count, average fixation duration, and total fixation duration between high- and low-skilled sim racers using independent *t*-tests.

To investigate whether high-skilled sim racers displayed greater overt attention allocation in corner sectors when metrics were adjusted for the lap time, we compared fixations per second, average, and total fixation durations as a proportion of corner sector time between high- and low-skilled sim racers using independent *t*-tests.

Sidak corrections were applied during the analyses as multiple *t*-tests were conducted for each hypothesis. The effect sizes are reported using *d*, and results as the means ± SD.

## 3. Results

### 3.1. Skill Level Comparisons

Higher- and lower-skilled sim racers significantly differed in the number of hours that they raced per week (*t*(31.690) = 5.567, *p* < 0.001, *d* = 1.666), with high-skilled sim racers spending 18.45 ± 10.43 h sim racing per week compared to the lower-skilled groups 4.42 ± 5.18 h (Table 1). Furthermore, high- and low-skilled sim racers’ fastest lap times were found to be significantly different (*t*(20.024) = −8.256, *p* < 0.001, *d* = 2.762), with high- and lower-skilled sim racers averaging lap times of 87,029.36 ms and 96,479.74 ms, respectively (Table 1). 

### 3.2. Raw Whole-Lap Fixation Metrics (Non-Normalised)

**Figure 3:**
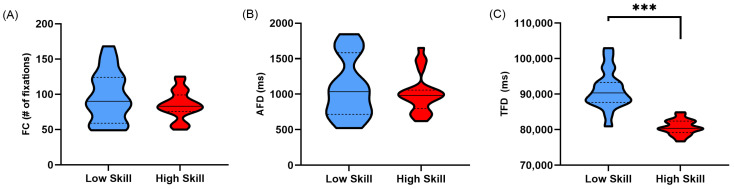
Truncated violin plots for non-normalised lap time fixation metrics; (**A**) fixation count (FC), (**B**) average fixation duration in milliseconds (AFD), and (**C**) total fixation duration in milliseconds (TFD) during participants’ fastest lap times. Data are presented as frequency distributions with medians (solid line) and quartiles (dashed lines) for lower-skilled (blue) and higher-skilled (red) sim racers. Significance (***) is denoted where *p* < 0.001.

Lower-skilled sim racers displayed a higher number of fixations (FC) over the course of their lap compared to high-skilled sim racers, although this was not significant (*t*(27.450) = −1.250, *p* = 0.111, *d* = 0.407) (Table 1; Figure 3A). Non-normalised average fixation duration (AFD) was found not to be significantly different between higher- and lower-skilled sim racers (*t*(29.869) = −0.675, *p* = 0.252, *d* = 0.218) (Table 1; Figure 3B). Finally, when examining total fixation duration (TFD), lower-skilled sim racers had a significantly higher total fixation duration than higher-skilled sim racers (*t*(22.412) = −7.879, *p* < 0.001, *d* = 2.661) (Table 1; Figure 3C).

### 3.3. Normalised Whole-Lap Fixation Metrics

**Figure 4:**
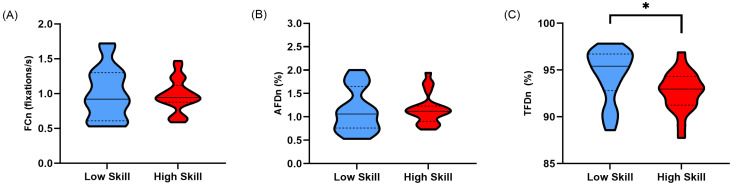
Truncated violin plots for lap-time-normalised fixation metrics; (**A**) fixations per second (FC_n_), (**B**) average fixation duration as a proportion of participants’ fastest lap time (AFD_n_), and (**C**) total fixation duration as a proportion of participants’ fastest lap time (TFD_n_). Data are presented as frequency distributions with medians (solid line) and quartiles (dashed lines) for lower-skilled (blue) and higher-skilled (red) sim racers. Significance (*) is denoted where *p* < 0.05.

When the fixation count was corrected for participants’ lap time (FC_n_), no significant difference was found between high- and low-skilled sim racers (*t*(29.917) = −0.229, *p* = 0.410, *d* = 0.074) (Table 1; Figure 4A). Normalising the average fixation duration (AFD_n_) resulted in no significant difference between higher- and lower-skilled sim racers (*t*(39) = 0.226, *p* = 0.411, *d* = 0.071) (Table 1, Figure 4B). Lastly, when normalising the total fixation duration (TFD_n_), a significant difference was still present, with lower-skilled sim racers fixating for a higher proportion of their lap time than higher-skilled sim racers (*t*(39) = −2.239, *p* = 0.013, *d* = 0.729) (Table 1; Figure 4C).

### 3.4. Fixation Allocation Ratio

**Figure 5:**
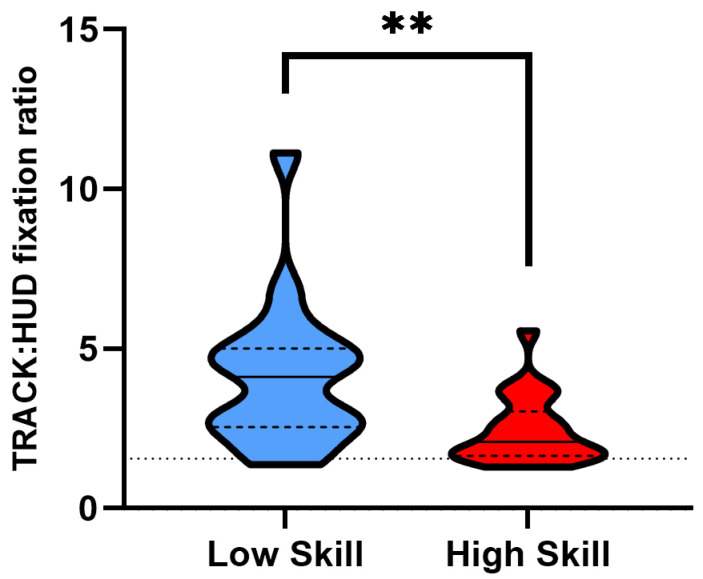
Truncated violin plots for ratio of on-track to HUD fixation count (TRACK:HUD). Data are presented as frequency distributions with medians (solid line) and quartiles (dashed lines) for lower-skilled (blue) and higher-skilled (red) sim racers. Dotted line represents the ratio of the AOI areas of TRACK to HUD. Significance (**) is denoted where *p* < 0.01.

When examining the TRACK:HUD fixation ratios, we found that low-skilled sim racers had a significantly higher ratio of their fixations on track in comparison to HUD elements when compared to high-skilled sim racers (*t*(24.618) = −2.946, *p* = 0.003, *d* = 0.969) (Table 1; Figure 5).

### 3.5. Raw Fixation Metrics in Corner Sectors (Non-Normalised)

**Figure 6:**
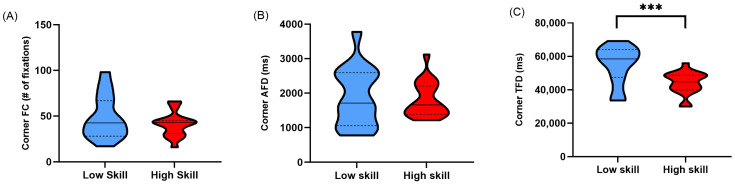
Truncated violin plots for non-normalised fixation metrics in corners; (**A**) fixation count (Corner FC), (**B**) average fixation duration in milliseconds (Corner AFD), and (**C**) total fixation duration in milliseconds (Corner TFD) during participants’ fastest lap times. Data are presented as frequency distributions with medians (solid line) and quartiles (dashed lines) for lower-skilled (blue) and higher-skilled (red) sim racers. Significance (***) is denoted where *p* < 0.001.

Lower-skilled sim racers displayed a higher number of fixations (Corner FC) when navigating corners when compared to higher-skilled sim racers, although this was not significant (*t*(22.639) = −1.129, *p* = 0.135, *d* = 0.401) (Table 2; Figure 6A). Non-normalised average fixation duration in corners (Corner AFD) was not found to be significantly different between lower- and higher-skilled sim racers (*t*(23.635) = −0.317, *p* = 0.377, *d* = 0.112) (Table 2; Figure 6B). Lastly, when examining non-normalised total fixation duration in corners (Corner TFD), a significant difference was found between lower- and higher-skilled sim racers (*t*(23.408) = −3.444, *p* < 0.001, *d* = 1.220) (Table 2; Figure 6C).

### 3.6. Normalised Fixation Metrics in Corner Sectors

**Figure 7:**
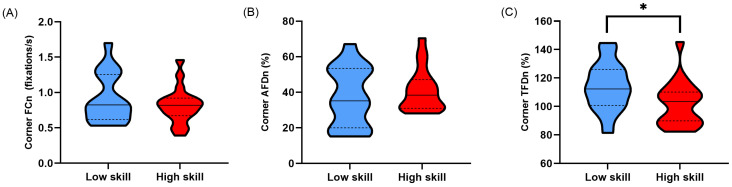
Truncated violin plots for normalised fixation metrics in corners; (**A**) fixations per second (Corner FC_n_), (**B**) average fixation duration as a proportion of participants’ corner time (Corner AFD_n_), and (**C**) total fixation duration as a proportion of participants’ corner time (Corner TFD_n_). Data are presented as frequency distributions with medians (solid line) and quartiles (dashed lines) for lower-skilled (blue) and higher-skilled (red) sim racers. Significance (*) is denoted where *p* < 0.05.

When the fixation count was normalised for the time spent in corners (Corner FC_n_), no significant difference was found between lower- and higher-skilled sim racers (*t*(33) = −0.964, *p* = 0.171, *d* = 0.327) (Table 2; Figure 7A). Normalising average fixation duration in corners (Corner AFD_n_) resulted in no significant difference between lower- and higher-skilled sim racers (*t*(33) = 0.814, *p* = 0.211, *d* = 0.276) (Table 2; Figure 7B). Finally, when the total fixation duration in corners was normalised for the time spent in corners (Corner TFD_n_), a significant difference was found between lower- and higher-skilled sim racers (*t*(33) = −2.181, *p* = 0.018, *d* = 0.740) (Table 2; Figure 7C).

## 4. Discussion

In this study, we set out to compare the visual attention of high- and low-skilled sim racers during a time trial task where participants tried to achieve their fastest lap time. By testing our first hypothesis, we found no significant difference in the fixation count or average fixation duration, although low-skilled sim racers had a significantly greater total fixation duration compared to high-skilled sim racers. When testing our second hypothesis, by correcting for the lap time duration of both groups, we found no significant difference in fixations per second or average fixation duration but did still see higher total fixation durations among lower-skilled compared to high-skill racers, contrary to expectations. When testing our third hypothesis, we found that low-skilled sim racers had a significantly greater ratio of fixations on-track compared to HUD elements than high-skilled sim racers, the opposite direction in relation to the hypothesis. Finally, in testing our fourth and fifth hypotheses relating to corner sectors of the lap, in line with our expectations, we found that low-skilled sim racers had a greater number of fixations and average fixation duration, although these were not significant, and a significantly greater total fixation duration than high-skilled sim racers. When correcting these metrics for corner sector time, similarly to the analysis of the whole lap, we found no significant difference in fixations per second or average fixation duration as a proportion of corner sector time across corner sectors, but against expectations, low-skilled sim racers displayed significantly greater total fixation durations as a proportion of corner sector time compared to high-skilled sim racers. We discuss the implications of our findings below.

As expected, low-skilled sim racers displayed greater overt attention allocation when their lap times were not corrected for across the whole lap and in corner sectors compared to high-skilled sim racers. This was evidenced by increased total fixation durations (Figure 3C and Figure 6C). Interestingly, this pattern of behaviour was maintained even after correcting for the increased temporal opportunity low-skilled sim racers had to allocate their attention over their slower lap and corner sector times (Figure 4C and Figure 7C). Much of the literature on self-paced tasks and visual behaviour has found that experts tend to fixate less and for longer duration compared to non-experts [13,14,15]. Our findings contradict this previous body of evidence by the fact that the fixation counts and average fixation durations were no different between our skill levels. However, in previous studies, data suggesting that experts fixate less frequently and for longer durations largely come from comparisons between groups with extreme differences in skill level (i.e., beginner/novice and expert/high skill). The groups in the current study may be more homogenous compared to those previous studies given that low-skilled sim racers still committed over 4 h per week to sim race training (Table 1).

Moreover, the fact that low-skilled sim racers displayed greater overt attention allocation than high-skilled sim racers suggests that a time trial sim racing task may more closely reflect an externally paced rather than a self-paced task. Externally paced tasks involve the recognition of meaningful cues, quick decision making, and the initiation of responses appropriate to the situation. Therefore, one must selectively attend to and quickly process information from only the most pertinent cues [26]. Examples of such cues include braking markers. As suggested by [27], what limits a driver’s speed is mostly the limits of the information processing of the brain. This may be why low-skilled sim racers display greater overt attention allocation, as they are taking longer to process information from these cues on track.

Overall, our findings evidence that higher-skill sim racers appear to need significantly less attention throughout their fastest lap and in corner sectors.

Contrary to our third hypothesis we note that low-skilled sim racers had a significantly higher ratio of fixations on track compared to high-skilled sim racers (Figure 5). That is not to say that either group overtly attended more to HUD elements relative to the track. This can be seen by the fact that the average fixation ratios of both groups fall above the ratio of the AOI areas of the two elements (see dotted line in Figure 5). Instead, we note that lower-skilled racers overtly attend relatively more to the track compared to HUD elements. This suggests that lower-skilled sim racers may not have the attentional resources to gather and utilise other on-screen information that may be relevant to higher levels of performance. Interestingly, a lower ratio of TRACK:HUD fixations for high-skilled sim racers did not hinder performance, as evidenced by their faster lap times. This potentially points to the importance of HUD elements for optimal sim racing performance. For example, lap time information, which contains current lap time versus best lap time (lap delta time), can inform where time may be gained throughout a racers lap. This pattern of overt attentional allocation among our high-skilled group can also be seen in research studying expertise in first-person shooter video games. Furukado and Hagiwara [28] found that high-skilled Valorant players attended significantly more to the in-game mini-map (giving the player HUD information on the locations of the opponents) than less skilled players. Thus, selective attention allocation may be an important feature of successful performance.

Despite this being the first study of visual attention in simulated racing, there are some limitations to the current study. There may be other eye movement parameters not examined in this study that are relevant to successful sim racing performance. Smooth/visual pursuit could be an important parameter while driving [29]. Unfortunately, given that our eye tracking device had a low sampling rate of 50 Hz, we could not accurately measure smooth pursuit movement. High precision data are needed to detect smooth pursuits [30] (p. 446). Secondly, although we are only focusing on overt attention allocation in the current study, it is possible that participants could have also been covertly attending. Higher-skilled sim racers may have had the ability to covertly attend to the track while they were overtly attending to HUD information, but this was not within the scope of the current study.

Although previous research has examined the driver head and eye movement in real [31] and virtual settings [32], the current study is the first to examine visual attention in a virtual racing setting. In doing so, we provide new insight into how sim racers of differing skill levels overtly allocate attention, which may be used for the talent identification and training of sim racing expertise. The implementation of gaze metrics has already been applied to training in surgery [33] and sporting domains [34,35]. As many highly skilled sim racers now transition into real-world racing, visual attention training could aid their performance. Eye movement variables that were not assessed in this study, such as saccades and gaze distribution, should be investigated in the future, as the total area of the scene being viewed may also be a fruitful avenue to explore expertise [36].

## 5. Conclusions

Overall, the findings of the current study differ from existing literature on visual behaviours and expertise, but provide explanations as to why sim racers of differing skill levels do show differences in their length of attention allocation. Furthermore, the importance of where overt attention is allocated is highlighted for successful sim racing performance. Thus, our findings contribute to a relatively new field of study, where much more research is needed to cognitively profile sim racers of differing expertise.

## Figures and Tables

**Table 1:** Summary of the hours spent sim racing and lap time, as well as non-normalised and normalised for lap time fixation metrics. Data expressed as the means, *SDs*, and *p*-values. Bolded *p*-values indicate a significant difference between values.

Variable	Low-Skilled (Mean ± SD)	High-Skilled (Mean ± SD)	*p*-Value
Hours sim racing/week	4.42 ± 5.18	18.45 ± 10.43	**<0.001**
Lap time (ms)	96,479.74 ± 4854.94	87,029.36 ± 1237.78	**<0.001**
Fixation count (FC)	96.58 ± 35.78	85 ± 20.85	0.111
Average fixation duration (AFD; ms)	1084.84 ± 423.88	1008.41 ± 272.35	0.252
Total fixation duration (TFD; ms)	91,183.47 ± 5482.65	80,681.41 ± 2069.5	**<0.001**
Fixations per second (FC_n_)	1.00 ± 0.36	0.98 ± 0.23	0.410
Normalised average fixation duration (AFD_n_; %)	1.13 ± 0.47	1.16 ± 0.32	0.411
Normalised total fixation duration (TFD_n_; %)	94.50 ± 2.8	92.71 ± 2.11	**0.013**
TRACK:HUD fixation ratio	4.11 ± 2.27	2.44 ± 1.06	**0.003**

**Table 2:** Summary for non-normalised and normalised corner sector time fixation metrics. Data are expressed as the means, SDs, and *p*-values. Bolded *p*-values indicate a significant difference between values.

Variable	Low-Skilled (Mean ± SD)	High-Skilled (Mean ± SD)	*p*-Value
Fixation count corners (Corners FC)	48.44 ± 23.45	41.00 ± 13.12	0.135
Average fixation duration corners (Corners AFD; ms)	1884.82 ± 854.70	1807.53 ± 511.96	0.372
Total fixation duration corners (Corners TFD; ms)	55,147.00 ± 11,210.09	44,170.42 ± 6614.49	**0.001**
Fixations per second corners (Corners FC_n_)	0.92 ± 0.35	0.82 ± 0.26	0.171
Normalised average fixation duration corners (Corners AFD_n_; %)	37.34 ± 16.62	41.30 ± 12.08	0.211
Normalised total fixation duration corners (Corners TFD_n_; %)	113.79 ± 16.95	101.70 ± 15.82	**0.018**

## Data Availability

Data can be obtained from M.J.C. via email request.

## Appendix A

In-game settings used for Assetto Corsa Competizione which were kept consistent for all participants.

Controls:

- Gain—80%
- Minimum Force—0%
- Damper—0%
- Dynamic Damping—100%
- Road Effects—40%
- Frequency—400Hz
- Steer Lock—1080°
- Steer Linearity—1.00
- Brake Gamma—1.00
- Gearshift Debouncing—50 ms
- Manufacturer Extras—Enabled
- Trueforce Audio—50%

Weather and Track Conditions:

- Dynamic Weather—Disabled
- Variability—0%
- Cloud Cover—25%
- Temperature—27 °C
- Random Weather—Disabled
- Grip Level—Fast
- Wetness—0%
- Standing Water—0%

Car Setup:

- Safe Preset
- Traction Control—6/12
- ABS—6/12
- Engine Map—1/12

Hardware settings used during testing which were kept consistent for all participants:

Logitech G Pro Wheel:

- Sensitivity—50
- Operating Range (angle)—1080
- Dampener—10
- Strength—8 Nm
- Force Feedback Filter—11

Logitech G Pro Pedals:

- Brake Sensitivity—50
- Throttle Sensitivity—50
- Brake Force—30 kg

## Appendix B

Below are the settings from the Tobii I-VT Attention filter used in the study.

Noise reduction—Moving median
Window size (samples)—3
Velocity calculator window length—20 ms
I-VT classifier—100 °/s
Merge adjacent fixations—On
Max. time between fixations—75 ms
Max. angle between fixations—0.5°
Discard short fixations—On
Minimum fixation duration—60 ms
Reclassify as saccade—Off
